# Correlation of metal ions with specific brain region volumes in neurodegenerative diseases

**DOI:** 10.55730/1300-0144.5714

**Published:** 2023-08-26

**Authors:** İsmet Murat MELEK, Berna KUŞ, Zülal KAPTAN, Emine PETEKKAYA

**Affiliations:** 1 Department of Neurology, Faculty of Medicine, Hatay Mustafa Kemal University, Hatay, Turkiye; 2 Department of Biochemistry, Faculty of Medicine, Hatay Mustafa Kemal University, Hatay, Turkiye; 3 Department of Physiology, Faculty of Medicine, Beykent University, İstanbul, Turkiye; 4 Department of Anatomy, Faculty of Medicine, Kastamonu University, Kastamonu, Turkiye

**Keywords:** Alzheimer’s disease, Parkinson’s disease, metal ions, voxel-based morphometry

## Abstract

**Background/aim:**

There are reports stating that deteriorations in metal homeostasis in neurodegenerative diseases promote abnormal protein accumulation. In this study, the serum metal levels in Alzheimer’s disease (AD) and Parkinson’s disease (PD) and its relationship with the cortical regions of the brain were investigated.

**Materials and methods:**

The patients were divided into 3 groups consisting of the AD group, PD group, and healthy control group (n = 15 for each). The volumes of specific brain regions were measured over the participants’ 3**-**dimensional magnetic resonance images, and they were compared across the groups. Copper, zinc, iron, and ferritin levels in the serums were determined, and their correlations with the brain region volumes were examined.

**Results:**

The volumes of left hippocampus and right substantia nigra were lower in the AD and PD groups, while the volume of the left nucleus caudatus (CdN) and bilateral insula were lower in the AD group compared to the control group. Serum zinc levels were lower in the AD and PD groups, while the iron level was lower in the PD group in comparison to the control group. In addition, the serum ferritin level was higher in the AD group than in the control group. Serum zinc and copper levels in the AD group were positively correlated with the volumes of the right entorhinal cortex, thalamus, CdN, and insula. Serum zinc and copper levels in the PD group showed a negative correlation with the left nucleus accumbens (NAc), right putamen, and right insula volumes. While the serum ferritin level in the PD group displayed a positive correlation with the bilateral CdN, putamen, and NAc, as well as the right hippocampus and insula volumes, no area was detected that showed a correlation with the serum ferritin level in the AD group.

**Conclusion:**

A relationship was determined between the serum metal levels in the AD and PD groups and certain brain cortical regions that showed volumetric changes, which can be important for the early diagnosis of neurodegenerative diseases.

## 1. Introduction

Alzheimer’s disease (AD) is a progressive neurodegenerative disease with high prevalence [1–3]. Changes such as oxidative stress, the metal ion imbalance, mitochondrial function disorder, and neuroinflammation have been investigated in relation to AD [4]. The accumulation of amyloid-beta (Aβ) clusters is among the differentiating properties of the disease [1–3, 5]. Among the regions where Aβ-loaded extracellular and intracellular plaques concentrate are the entorhinal cortex, hippocampus, amygdala, and limbic system structures [2]. The neuropathology of AD is also characterized by the formation of neurofibrillary tangles that are made up of hyperphosphorylated tau protein and cortical neuronal loss [1, 3]. Patients with AD typically display amnestic memory loss, and deterioration is observed in other mental abilities and daily living activities as the disease progresses [1]. Recent studies have shown that one of the potential risk factors for dementia is the age-related imbalance of metal homeostasis [1–9]. Normally, metal homeostasis is strictly controlled, but any disorder results in severe problems [7]. On the other hand, it can contribute to the disease, and possibly aging, by activating or inhibiting enzymatic reactions, increasing reactive oxygen species (ROS) types, competing with other elements, and affecting permeability of cell membranes [7]. This approach, which addresses the role of metals in neurodegenerative diseases, assumes the failure of endogen regulating mechanisms that lead to metal imbalance, rather than toxic exposure to metals [9].

In the clinical diagnosis of AD, a detailed history regarding the type and progress of the symptoms is taken from the patient or another source (e.g., caregiver) in order to determine whether cognitive deterioration is present, and a neuropsychological evaluation is made [3, 5]. There are studies on the use of imaging techniques, such as magnetic resonance imaging (MRI), functional MRI, and positron emission tomography (PET), in AD diagnosis and their importance in evaluating brain pathology in AD, but these approaches are not routinely used [3, 5]. Atrophy measurement in certain structures is actively used in identifying patients with mild cognitive disorders that will progress towards AD [5]. On the other hand, atrophy differences between dementia patients with AD and without AD are less definite. Many studies are being conducted on cerebrospinal fluid (CSF) and serum indicators that reflect AD pathology [3, 10, 11]. As mentioned earlier, it seems that the imbalance of metals in the human body can be a potential determinant factor for neurodegenerative diseases. Therefore, in order to offer effective and novel therapeutic approaches, it might also be important to determine the changes in the metal levels that occur in these diseases.

Although serum samples have a disadvantage because they do not directly provide information about metal status of the brain, since it is possible to collect them from more patients and with easier methods, establishing a connection between changes in the serum metal levels and AD and other neurodegenerative disorders will prove beneficial. The amyloid cascade hypothesis, which states that the central event in AD pathology is the accumulation of amyloid-beta (Aβ) peptide in the brain, assumes that Aβ passes through the membrane and aggregates to amyloid plaques when copper (Cu^2+^), iron (Fe^3+^), and zinc (Zn^2+^) have highly accumulated [2]. Normally, the metal ion content of the brain is strictly regulated, and passive metal flow from the circulation to the brain does not occur; in other words, the passing of metals over the blood-brain barrier (BBB) is strictly regulated [8, 9]. However, considering that this control mechanism is disrupted in neurodegenerative diseases, and there are disruptions that occur in homeostatic mechanisms that separate the metals into compartments and regulate them, examining metal levels in the serum is also important. Hence, while the serum metal levels in AD and Parkinson’s disease (PD) patients were analyzed in this study, it was aimed to examine the changes here along with the atrophies in specific regions of the brain.

## 2. Materials and methods

### 2.1. Volunteer selection

The study was conducted on AD and PD patients who presented to the Neurology Clinic of the Faculty of Medicine at Hatay Mustafa Kemal University as well as healthy individuals. Included in the study were 15 AD patients with mild to moderate cognitive disorders, 15 PD patients, and 15 healthy individuals (8 females and 7 males in each group), with 45 voluntary participants in total. The AD patients were volunteers with mild cognitive disorders who were diagnosed with the disease according to the National Institute of Neurological and Communicative Disorders and Stroke/Alzheimer’s Diseases and Related Disorders Association criteria. The PD patients were diagnosed according to The Parkinson’s United Kingdom Brain Bank criteria. Patients who had mild PD (H&Y stages I–II) according to the Hoehn-Yahr staging criteria (United Parkinson’s Disease Rating Scale) were included in the study. The participant groups were formed using the simple random sampling method among patients who applied to the Hatay Mustafa Kemal University Neurology Clinic. Patients with AD and PD who were physically able to stay in the magnetic resonance imaging (MRI) device for at least 10 min were included in the study, while patients whose mobility and mental orientation did not allow this were excluded from the study. The control group consisted of individuals who had similar age and educational characteristics but did not have a diagnosed brain pathology or any symptoms that would suggest the possibility of a brain pathology. Individuals who had brain trauma, brain tumors, seizures, or clinical histories accompanied by other psychological symptoms, heart diseases, and diabetes, and who smoked were excluded from the study. Informed consent of the participants was obtained. Ethical approval for the study was received from the Hatay Mustafa Kemal University Clinical Research Ethics Committee (Approval number: 4298783/050-35, Dated: 15.09.2020).

### 2.2. Voxel-based morphometry

High resolution, 3-dimensional (3D) axial brain magnetic resonance (MR) images of the participants were obtained. For volumetric analysis of the brain and certain brain lobar segments of the groups, MATLAB 7.10.0 software, which includes the Statistical Parametric Mapping (SPM12) application was used with a combined segmentation approach. After the images were segmented into gray matter (GM), white matter (WM), and CSF using the CAT12 toolbox in MATLAB (MathWorks, Natick, MA, USA). The images were then normalized to the Montreal Neurological Institute (MNI) space1 standard. Thus, the images were aligned by correcting for differences in the participants’ head positions or orientation during scanning. Normalized images were removed from the noise effect during the smoothing phase. Finally, the volumes of the regions of interest were calculated through the atlas-based region of interest (ROI) analysis. Although some studies have found differences in manual measurements compared to automated measurement, the atlas-based automated measurement is a very valid method in the literature [12].

### 2.3. Biochemical analysis of the blood samples

In the morning, following a 12-h fast, 5 cc of venous blood samples were taken from the left forearm of the volunteers, the samples were centrifuged at the Hatay Mustafa Kemal University Central Laboratory, and their serums were separated. Serum copper and iron levels were analyzed through atomic absorption assay (AAS). Since atoms can absorb light of a specific wavelength, when that specific wavelength is provided, the light is absorbed by that atom. In this method, the concentration of the element is calculated according to the amount of light absorbed. Serum zinc levels were measured using the colorimetric method with an Archem Zn kit (Archem, Diagnostics, Bağcılar, İstanbul, Türkiye) on an Atellica CH 930 autoanalyzer (Siemens Healthineers, Erlangen, Germany). In this method, when the kit reactant reacts with zinc, it forms a complex that absorbs at 546 nm. Ferritin levels were studied on an Advia Centaur XP hormone analyzer (Siemens Healthineers). This is a 2-step sandwich immunoassay using fixed amounts of 2 antiferritin antibodies using direct chemiluminometric technology.

### 2.4. Statistical analysis

The study data were analyzed using SPSS Statistics for Windows 16.0 (SPSS Inc., Chicago, IL, USA) with a 95% confidence interval. Considering the number of volunteers and their distribution characteristics (did not show normal distribution according to the Shapiro-Wilk test), in the comparison of volumes of brain regions and serum metal levels of the groups, the nonparametric Kruskal-Wallis test was used, followed by the Dunn test as a post hoc test. The Spearman rank correlation coefficient was used for the correlation analysis. The Wilcoxon test was used to compare brain areas between the right and left hemispheres. Statistical significance was accepted as p < 0.05.

## 3. Results

### 3.1. Demographic characteristics

The mean age was 74.50 **±** 8.01 years in the AD group, 73.43 **±** 5.86 years in the PD group, and 73.00 **±** 6.73 years in the control group. No statistically significant difference was determined between the groups in terms of sex distribution.

### 3.2. Volumetric comparisons

**Figure 1:**
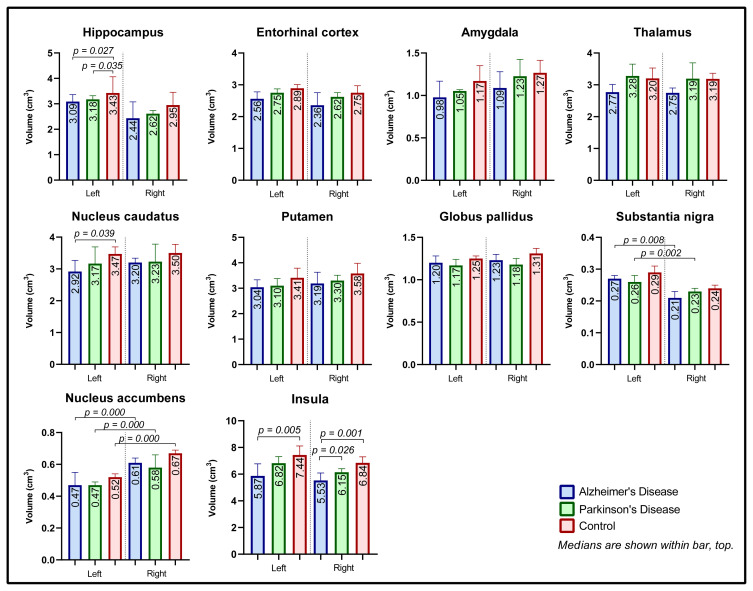
Volumes of the brain areas.

Median volumes of the examined brain regions for each group and their comparisons are presented in Figure 1 and Table 1. The left hippocampus volume in the AD and PD groups was significantly lower compared to the control group (p = 0.027 and p = 0.035, respectively). A significant difference was found between the left and right hippocampus volumes in the AD and PD groups (p = 0.005 and p = 0.002, respectively). No difference was determined between the groups in the right hippocampus, bilateral amygdala, entorhinal cortex, or thalamus.

The left nucleus caudatus volume was significantly decreased in the AD group compared to the control group (p = 0.039). The putamen and globus pallidus volumes did not differ in the AD, PD, and control groups. In both the AD and PD groups, the right substantia nigra volume was lower compared to the left (p = 0.008 and p = 0.002, respectively). The left nucleus accumbens had a lower volume on the right side in all of groups (p = 0.000).

Both the left and right insula volumes were lower in the AD group than in the control group (p = 0.005 and p = 0.001, respectively). The right insula volume in the AD group was also lower compared to the control group (p = 0.026).

### 3.3. Metal ions and ferritin

**Figure 2:**
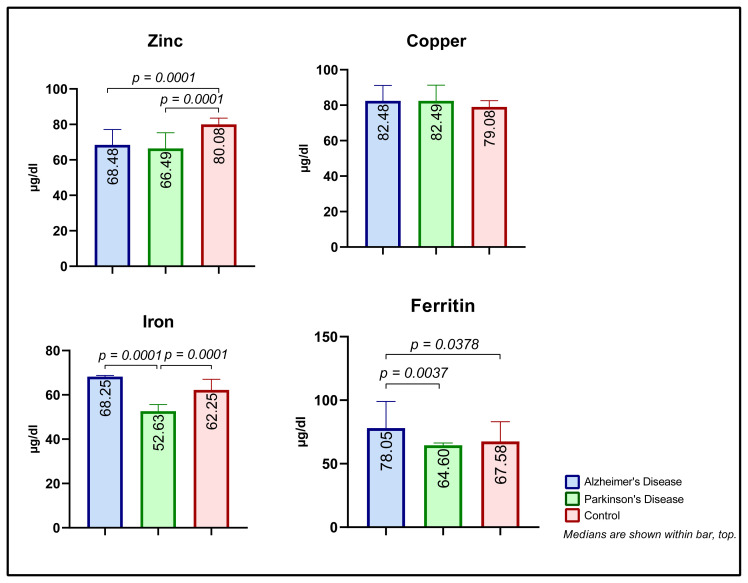
Serum metal and ferritin levels.

The median values of the serum metal levels of the groups and their comparisons are presented in Figure 2 and Table 2. The iron level in the serum in the PD group was lower than in both the AD and control groups (p = 0.000). The serum zinc level was significantly lower both in the AD and PD groups compared to the control group (p = 0.000). No significant difference was found between the groups in terms of the serum copper levels. As for the serum ferritin levels, it was higher in the AD group in comparison to both the PD and control group (p = 0.004 and p = 0.038, respectively).

### 3.4. Correlations

Serum zinc levels in the AD group were positively and strongly correlated with the right entorhinal cortex, right thalamus, right nucleus caudatus, and right insula (p = 0.037, p = 0.035, and p = 0.042, respectively) and positively and moderately correlated with the right thalamus (p = 0.032). Serum copper levels in the AD group were positively and moderately correlated with the right entorhinal cortex, right thalamus, right nucleus caudatus, and right insula (p = 0.041, p = 0.044, p = 0.045, and p = 0.050, respectively) (Table 3).

A negative and moderate correlation was found between the serum zinc levels in the PD group, in the left nucleus accumbens, right putamen, and right insula (p = 0.025, p = 0.040, and p = 0.030, respectively). Serum copper levels were also negatively and moderately correlated with the left nucleus accumbens, right putamen, and right insula (p = 0.020; p = 0.034, and p = 0.028, respectively) (Table 4).

Serum ferritin levels in the PD group showed a positive and moderate correlation with the left hippocampus and right insula volumes (p = 0.041 and p = 0.027, respectively). A positive and strong correlation was also found between the left globus pallidus volume and serum ferritin level (p = 0.010). The serum ferritin level also displayed a very strong and positive correlation with left nucleus caudatus volume (p = 0.000) and a moderate and positive correlation with right nucleus caudatus volume (p = 0.005). A positive and strong correlation was determined between the left and right putamen volumes and serum ferritin levels in the PD group (p = 0.002 for both). The left and right nucleus accumbens volumes displayed a strong and positive correlation with the serum ferritin level (p = 0.002 and p = 0.005, respectively) (Table 4). Serum ferritin levels in the AD group did not show any correlations with the examined brain region volumes (Table 3).

In the control group, the serum ferritin levels showed moderate and strong negative correlations with the left amygdala (p = 0.016) and insula (p = 0.025) volumes, respectively. Zinc and copper levels showed a very strong positive correlation with the left putamen volume and right nucleus accumbens volume (p = 0.000 for each) (Table 5).

## 4. Discussion

Metal ions such as iron, zinc, and copper are called neurometals, due to their important roles in maintaining the functions of the central nervous system [13]. Metal deficiency causes damage to the central nervous system [14]. On the other hand, excess levels of metals are also highly neurotoxic [15]. Homeostatic disturbances of metals contribute to the pathogenesis of many neurodegenerative diseases [14]. Thus, approaches to restore metal homeostasis may be a therapeutic target in neurodegenerative diseases. For this reason, research on how metal homeostasis is impaired in neurodegenerative diseases and how this deterioration can be detected should increase. In this study, the relationships between certain cortical area volumes and serum metal levels in AD and PD patients were investigated. Thus, it was attempted to verify the power of 2 diagnostic tools that have not yet been used routinely.

As a result, while some areas shared atrophic features in the AD and PD groups, differences were observed in some other areas. For example, while the AD group showed specific atrophic features, mostly in the medial temporal lobe areas, the atrophies became more evident in the basal nuclei group in the PD group. The metal ions showed differences in these diseases compared to the control group. While zinc levels were high in the control group, relatively decreased values were found in the AD and PD groups. In the PD group, serum zinc and copper levels were moderately negatively correlated with the left nucleus accumbens, right putamen, and also right insula volumes among the basal nuclei group. Zinc and copper levels in the AD group showed a strong positive correlation with the right entorhinal cortex and right insula, which are primary olfactory and taste areas and the right thalamus, as a sensory integration center, and also with the right nucleus caudatus volume as in the PD group.

As the catalytic component of many enzymes, copper is involved in essential processes such as energy metabolism, antioxidant defense, iron metabolism, and neurotransmitter synthesis, and plays a significant role in normal development and functioning of the brain [13]. In the literature, higher serum copper levels in the AD group compared to the control group have been reported [16, 17], and this has been associated with a faster progression of dementia [18]. Zinc is strongly bonded to proteins and peptides, and it is particularly important for appropriate protein folding [4] and can have pathological effects in neurodegenerative disorders, including AD [8, 9]. Depending on decreased antioxidant defenses and/or increased ROS and free radical production, an increase in zinc can be seen in the brain [8]. In fact, when postmortem AD brains were examined, differences in the levels of proteins and carriers included in zinc homeostasis were determined [4]. These are also related with the progress of the disease and severity of cognitive disorder [4]. Furthermore, serum zinc levels were found to be decreased in the AD group compared to the control group [19]. In the study they conducted, Baum et al. [6] also observed a decrease in serum zinc levels in the AD group, and they reported that this might have resulted from the accumulation of zinc in the brain amyloid and maybe from the exhaustion of zinc in other parts of the body. Zinc that is expressed in neocortical glutamatergic synapses is in interaction with plasma [20]. This view was supported by a study which demonstrated that the plasma zinc level in AD patients was lower than that in the control group, but that after treatment with zinc-binding combined clioquinol, it significantly increased and reached the normal range, and that the amyloid was broken down as a result of the treatment, and the zinc coming from that contributed to improving the serum zinc level [21].

These reports are consistent with the findings of the current study. In addition, the findings herein indicated that the serum levels of these 2 metals are positively correlated with the right entorhinal cortex, insula, thalamus, and nucleus caudatus in the AD group. It should be noted that all of these structures were smaller than those in the control group, although statistical significance was not found. The serum zinc level decreased and copper level increased in the AD group compared to the control group, both of which were positively correlated with the volumes of the brain areas. The serum zinc level decreased and copper level increased in the PD group compared to the control group, but both showed a negative correlation with the volumes of the atrophic brain areas. From this it is seen that primarily zinc and copper act in different directions. Thus, in a study that examined zinc, copper, and manganese levels among metal ions in the anatomical structures of the aged brain, Ramos et al. [22] determined the highest copper and manganese levels in the putamen (representing the PD group in the present study) and the highest zinc levels in hippocampus and medial temporal lobe regions (representing the AD group in the present study). These studies were not comprehensive enough to explain the causality. The homeostases of these 2 metals need to be studied with molecular techniques. The speculation that can be made according to what has been seen for now is as follows: In PD, the increased copper in the serum may also reflect on the putamen and cause atrophy (which appears as a negative correlation), and in AD, the decreased zinc in the serum may accumulate in the medial temporal lobe structures and cause atrophy (which appears as a positive correlation).

In both cases, considering previous studies, we predict that the accumulation of metals in the brain tissue is effective in neurodegenerative processes. The amyloid hypothesis proposes that Aβ aggregation triggers a series of events that lead to oxidative stress, neuronal function disorder, deteriorated plasticity, and apoptosis [2]. In this process, it is assumed that Aβ passes through the membrane and aggregates to amyloid plaques in the presence of a high accumulation of Cu^2+^, Fe^3+^, and Zn^2+^ [2]. In fact, amyloid plaques in postmortem brains with AD (especially in hippocampus, amygdala, and some regions of the cortex) include metals such as Zn^2+^, Cu^2+^, and Fe^3+^ [2]. The role played by the aforementioned metals in the cause-effect chain in AD has not yet been clarified.

In the present study, the highest serum ferritin level was observed in the AD group, while the lowest was found in the PD group. In addition to Zn^2+^ and Cu^2+^, Fe^3+^ can also induce Aβ peptide aggregation in AD [3, 9, 23]. Studies that examined postmortem brain tissue samples reported gradually increasing iron levels in the brains of AD patients compared to the control group [2, 9]. Iron homeostasis mostly changes in neurodegenerative disorders [2, 10, 24]; under certain conditions, this metal is the strongest prooxidant due to its high presence [9, 25]. Excessive ROS production oxidates proteins, DNA, and phospholipids and leads to structural and functional changes [26]. Ferritin is basically an intracellular iron storage protein itself, and serum ferritin generally reflects iron storages in the liver [3, 27]. It shows greater increase in microglia and senile plaques [2, 27]. Moreover, serum ferritin is an acute phase indicator, and therefore, it is also an indicator of inflammation [27]. The increase observed in the serum ferritin levels in the AD group can be attributed to the leakage from the brain due to deterioration in the integrity of the BBB [28]. However, as infection acute phase reactants were not analyzed in the current study, it is not possible to mention it as an inflammation marker. In the present study, the serum iron level in the AD group did not show a difference compared to the control group. On the other hand, it was lower in the PD group compared to both the AD and control groups. Some studies conducted have reported an increase in zinc and iron and a decrease in copper in the substantia nigra in the PD group [9, 29]. Previously, as mentioned above, these changing levels in the serum may have resulted from the aggregation of metals in certain regions.

Therapeutic approaches targeting metal homeostasis in neurodegenerative diseases have aimed to restore neural metal content through the administration of metals or the use of metal chelators [14]. Previous studies have reported that zinc administration can improve neurological functions in AD patients [19]. Clioquinol, the triple chelator of iron, copper, and zinc, has been shown to reduce cognitive deficits in AD patients [30], although its clinical limitations were later revealed [14]. Ultimately, however, simply supplementing or chelating metals can cause side effects [14]. In conclusion, it is essential to elucidate metal metabolic mechanisms in order to target metals in the treatment of neurodegenerative diseases. Thus, ion channels can also be targeted to regulate metal homeostasis. In addition, recent research has shown that approaches to iron-related lipid peroxidation may be therapeutic for AD and PD patients [14].

### 4.1. Conclusion

Although this study was preliminary and had important limitations, it is our belief that it points to the changes in serum metal levels that should be focused on as specific to the 2 most common neurodegenerative diseases.

One of the 2 most important limitations of this study was the relatively small sample size. However, when considering the information obtained from the latest review [14], it is believed that the statistical differences determined herein are also clinically significant.

In addition, patients with PD dementia were not included in this study. Dementia is seen in 30% of PD patients [31]. In this context, it may be useful to compare PD and AD dementia with serum metal levels.

Although the data obtained on the effects of metal ions in this study did not provide evidence through brain tissue or CSF examinations and molecular signaling pathways, it is hoped that these bioindicators will be beneficial in terms of especially early diagnosis of AD and PD and the transformation of mild cognitive disorder into AD with the contribution of future studies.

## Figures and Tables

**Table 1:** Comparison of volumes of brain areas between the groups and hemispheres.

	Hippocampus (cm^3^)			Entorhinal cortex (cm^3^)			Amygdala (cm^3^)			Thalamus (cm^3^)			Nucleus caudatus (cm^3^)		
Group	Left	Right	p-value	Left	Right	p-value	Left	Right	p-value	Left	Right	p-value	Left	Right	p-value
AD	3.09	2.44	0.005	2.56	2.36	0.183	0.98	1.09	0.067	2.77	2.75	0.684	2.92	3.20	0.644
PD	3.18	2.62	0.002	2.75	2.62	0.375	1.05	1.23	0.102	3.28	3.19	0.627	3.17	3.23	0.117
Control	3.43	2.95	0.061	2.89	2.75	0.200	1.17	1.27	0.765	3.2	3.19	0.204	3.47	3.50	0.816
Comparison Between Groups p-value	AD-Control: 0.027 PD-Control: 0.035 AD-PD: 1.000	0.540		0.103	0.340		0.320	0.112		0.098	0.340		AD-Control: 0.038 PD-Control: 0.918 AD-PD: 0.540	0.430	
	Putamen (cm^3^)			Globus pallidus (cm^3^)			Substantia nigra (cm^3^)			Nucleus accumbens (cm^3^)			Insula (cm^3^)		
Group	Left	Right	p-value	Left	Right	p-value	Left	Right	p-value	Left	Right	p-value	Left	Right	p-value
AD	3.04	3.19	0.224	1.20	1.23	0.105	0.27	0.21	0.000	0.47	0.61	0.000	5.87	5.53	0.113
PD	3.10	3.30	0.705	1.17	1.18	0.923	0.26	0.23	0.000	0.47	0.58	0.000	6.82	6.15	0.172
Control	3.41	3.58	0.829	1.25	1.31	0.330	0.29	0.24	0.000	0.52	0.67	0.000	7.44	6.84	0.342
Comparison Between Groups p-value	0.391	0.383		0.148	0.114		0.350	0.104		0.255	0.103		AD-Control: 0.005 PD-Control: 1.000 AD-PD: 0.755	AD-Control: 0.001 PD-Control: 1.000 AD-PD: 0.026	

Values are expressed as the median.

**Table 2:** Comparison of the serum metal levels between the groups.

Group	Zinc (μg/dL)	Copper (μg/dL)	Iron (μg/dL)	Ferritin (μg/dL)
**AD**	68.48	82.48	68.25	78.05
**PD**	66.49	82.49	52.63	64.6
**Control**	80.08	79.08	62.25	67.58
**p-value**	AD-Control: 0.000 PD-Control: 0.000 AD-PD: 1.000	0.177	AD-Control: 0.131 PD-Control: 0.000 AD-PD: 0.000	AD-Control: 0.038 PD-Control: 1.000 AD-PD: 0.004

Values are expressed as the median.

**Table 3:** Correlation of the brain area volumes and serum metal levels in the AD group.

Left side of the brain		Ferritin	Iron	Zinc	Copper	Right side of the brain		Ferritin	Iron	Zinc	Copper
Hippocampus	r	−0.21	−0.12	0.00	0.00	Hippocampus	r	−0.02	0.26	0.38	0.38
	p	0.54	0.72	0.99	0.99		p	0.96	0.45	0.25	0.25
Amygdala	r	−0.01	−0.15	−0.42	−0.42	Amygdala	r	0.30	0.21	0.29	0.29
	p	0.98	0.67	0.20	0.20		p	0.37	0.53	0.39	0.39
Entorhinal cortex	r	−0.50	0.13	−0.13	−0.13	Entorhinal cortex	r	0.51	0.31	**0.695** ^**^	**0.633** ^**^
	p	0.12	0.69	0.71	0.71		p	0.11	0.35	**0.037**	**0.041**
Thalamus	r	−0.11	−0.02	−0.45	−0.45	Thalamus	r	−0.20	−0.05	**0.614** ^**^	**0.552** ^**^
	p	0.76	0.96	0.16	0.16		p	0.55	0.88	**0.032**	**0.044**
Nucleus caudatus	r	−0.36	−0.01	−0.47	−0.47	Nucleus caudatus	r	0.17	0.02	**0.745** ^**^	**0.637** ^**^
	p	0.27	0.98	0.15	0.15		p	0.61	0.96	**0.035**	**0.045**
Putamen	r	−0.35	−0.01	−0.45	−0.45	Putamen	r	−0.13	−0.02	−0.47	−0.47
	p	0.29	0.98	0.16	0.16		p	0.70	0.96	0.14	0.14
Globus pallidus	r	−0.09	−0.02	−0.47	−0.47	Globus pallidus	r	−0.18	−0.01	−0.47	−0.47
	p	0.80	0.96	0.14	0.14		p	0.59	0.98	0.15	0.15
Substantia nigra	r	−0.19	0.16	−0.26	−0.26	Substantia nigra	r	−0.22	−0.01	−0.49	−0.49
	p	0.57	0.63	0.43	0.43		p	0.52	0.98	0.13	0.13
Nucleus accumbens	r	−0.02	−0.08	−0.47	−0.47	Nucleus accumbens	r	−0.26	0.05	−0.53	−0.53
	p	0.95	0.82	0.14	0.14		p	0.45	0.89	0.09	0.09
Insula	r	−0.13	−0.02	−0.45	−0.45	Insula	r	−0.27	0.03	**0.825** ^**^	**0.602** ^**^
	p	0.71	0.96	0.16	0.16		p	0.43	0.93	**0.042**	**0.050**

r: Correlation coefficient, p: p-value.

**Table 4:** Correlation of the brain area volumes and serum metal levels in the PD group.

Left side of the brain		Ferritin	Iron	Zinc	Copper	Right side of the brain		Ferritin	Iron	Zinc	Copper
Hippocampus	r	0.533*	−0.05	−0.51	−0.51	Hippocampus	r	0.31	−0.22	−0.21	−0.21
	p	**0.041**	0.86	0.05	0.05		p	0.26	0.43	0.45	0.45
Amygdala	r	0.36	−0.08	−0.31	−0.31	Amygdala	r	0.34	0.05	−0.40	−0.40
	p	0.19	0.77	0.26	0.26		p	0.22	0.87	0.14	0.14
Entorhinal cortex	r	−0.25	−0.01	−0.19	−0.19	Entorhinal cortex	r	−0.20	0.01	−0.18	−0.18
	p	0.37	0.97	0.50	0.50		p	0.47	0.97	0.51	0.51
Thalamus	r	−0.14	−0.01	−0.19	−0.19	Thalamus	r	−020	0.01	−0.18	−0.18
	p	0.62	0.97	0.50	0.50		p	0.48	0.97	0.51	0.51
Nucleus caudatus	r	0.996**	0.13	−0.50	−0.50	Nucleus caudatus	r	0.682**	0.35	−0.33	−0.33
	p	**0.000**	0.64	0.06	0.06		p	**0.005**	0.20	0.22	0.22
Putamen	r	0.725***	0.30	−0.39	−0.39	Putamen	r	0.724**	−0.17	**−0.532** ^*^	**−0.562** ^*^
	p	**0.002**	0.28	0.15	0.15		p	**0.002**	0.54	**0.04**	**0.034**
Globus pallidus	r	−0.44	0.21	0.32	0.32	Globus pallidus	r	0.643**	−0.20	−0.46	−0.46
	p	0.10	0.46	0.24	0.24		p	**0.010**	0.48	0.08	0.08
Substantia nigra	r	−0.08	−0.10	−0.46	−0.46	Substantia nigra	r	0.19	0.28	−0.20	−0.20
	p	0.77	0.72	0.09	0.09		p	0.51	0.31	0.48	0.48
Nucleus accumbens	r	0.735**	−0.19	**−0.554** ^*^	**−0.579** ^*^	Nucleus accumbens	r	0.686**	−0.17	−0.48	−0.48
	p	**0.002**	0.50	**0.025**	**0.02**		p	**0.005**	0.53	0.07	0.07
Insula	r	0.30	−0.01	−0.36	−0.36	Insula	r	0.569*	−0.16	**−0.550** ^*^	**−0.570** ^*^
	p	0.29	0.98	0.18	0.18		p	**0.027**	0.58	**0.03**	**0.028**

r: Correlation coefficient. p: p-value.

**Table 5:** Correlation of the brain area volumes and serum metal levels in the control group.

Left side of the brain		Ferritin	Iron	Zinc	Copper	Right side of the brain		Ferritin	Iron	Zinc	Copper
Hippocampus	r	−0.20	0.78	0.40	0.40	Hippocampus	r	0.05	0.13	0.35	0.35
	p	0.48	0.78	0.14	0.14		p	0.85	0.65	0.20	0.20
Amygdala	r	−0.610*	0.17	0.46	046	Amygdala	r	−0.51	0.11	0.45	0.45
	p	**0.016**	0.55	0.09	0.09		p	0.05	0.69	0.09	0.09
Entorhinal cortex	r	0.29	0.13	0.11	0.11	Entorhinal cortex	r	0.04	−0.24	−0.30	−0.30
	p	0.30	0.64	0.69	0.69		p	0.89	0.38	0.28	0.28
Thalamus	r	0.29	0.13	0.13	0.13	Thalamus	r	−0.38	0.48	−0.14	−0.14
	p	0.30	0.54	0.66	0.66		p	0.89	0.07	0.61	0.61
Nucleus caudatus	r	−0.21	0.37	0.49	0.49	Nucleus caudatus	r	−0.21	0.10	0.49	0.49
	p	0.45	0.72	0.54	0.54		p	0.45	0.72	0.79	0.79
Putamen	r	−0.21	0.12	0.997**	0.997**	Putamen	r	−0.21	0.12	0.41	0.41
	p	0.45	0.68	**0.000**	**0.000**		p	0.45	0.68	0.19	0.19
Globus pallidus	r	−0.48	0.01	−0.10	−0.10	Globus pallidus	r	−0.19	−0.21	−0.42	−0.42
	p	0.06	0.96	0.74	0.74		p	0.50	0.46	0.12	0.12
Substantia nigra	r	−0.33	0.03	0.39	0.39	Substantia nigra	r	−0.41	0.04	0.42	0.42
	p	0.24	0.93	0.16	0.16		p	0.13	0.90	0.12	0.12
Nucleus accumbens	r	−0.19	0.08	0.10	0.10	Nucleus accumbens	r	−0.16	0.06	0.991**	0.991**
	p	0.49	0.78	0.33	0.33		p	0.56	0.84	**0.000**	**0.000**
Insula	r	−0.575*	0.15	0.19	0.19	Insula	r	−0.47	0.27	0.23	0.23
	p	**0.025**	0.59	0.50	0.50		p	0.08	0.33	0.41	0.41

r: Correlation coefficient. p: p-value.
